# Molecular Evolution of the *Fusion* (*F*) Genes in Human Parainfluenza Virus Type 2

**DOI:** 10.3390/microorganisms13020399

**Published:** 2025-02-12

**Authors:** Tatsuya Shirai, Fuminori Mizukoshi, Ryusuke Kimura, Rina Matsuoka, Mitsuru Sada, Kazuya Shirato, Haruyuki Ishii, Akihide Ryo, Hirokazu Kimura

**Affiliations:** 1 Department of Virology III, National Institute of Infectious Diseases, Musashimurayama-shi 208-0011, Tokyo, Japan; shirai@niid.go.jp (T.S.); mzksh@niid.go.jp (F.M.); shirato@niid.go.jp (K.S.); 2 Department of Respiratory Medicine, Faculty of Medicine, Kyorin University, Mitaka-shi 181-8611, Tokyo, Japan; rainbow_orch@ks.kyorin-u.ac.jp (M.S.); h141@ks.kyorin-u.ac.jp (H.I.); 3 Advanced Medical Science Research Center, Gunma Paz University, Takasaki-shi 370-0006, Gunma, Japan; 4 Department of Bacteriology, Graduate School of Medicine, Gunma University, Maebashi-shi 371-8511, Gunma, Japan; m2220015@gunma-u.ac.jp; 5 Department of Health Science, Graduate School of Health Sciences, Gunma Paz University, Takasaki-shi 370-0006, Gunma, Japan; r-matsuoka@paz.ac.jp

**Keywords:** human parainfluenza virus type 2, fusion gene, molecular evolution, conformational epitopes

## Abstract

Human parainfluenza virus type 2 (HPIV2) is a clinically significant respiratory pathogen, which highlights the necessity of studies on its molecular evolution. This study investigated the evolutionary dynamics, phylodynamics, and structural characteristics of the HPIV2 fusion (*F*) gene using a comprehensive dataset spanning multiple decades and geographic regions. Phylogenetic analyses revealed two distinct clusters of HPIV2 *F* gene sequences, which were estimated to have diverged from a common ancestor approximately a century ago. Cluster 1 demonstrated a higher evolutionary rate and genetic diversity compared to the more stable cluster 2. Bayesian Skyline Plot analyses indicated a significant increase in the effective population size of the *F* gene between 2005 and 2015; potentially linked to enhanced diagnostic and surveillance capabilities. Structural modeling identified conserved conformational epitopes predominantly in the apex and stalk regions of the F protein. These findings underscore the evolutionary constraints and antigenic landscape of the HPIV2 F protein.

## 1. Introduction

Human parainfluenza virus (HPIV) belongs to the Paramyxoviridae family and is classified as a negative-sense, single-stranded RNA virus. HPIVs are categorized into four distinct serotypes—HPIV1, HPIV2, HPIV3, and HPIV4—based on genetic and antigenic variations [1]. Among these, HPIV1 and HPIV3 are grouped within the genus *Respirovirus*, whereas HPIV2 and HPIV4 are classified under the genus *Rubulavirus* [1]. HPIVs are distributed worldwide and contribute to various respiratory diseases. However, their circulation patterns are influenced by geography and seasonality [2,3,4,5]. For instance, in the United States, HPIV1 and HPIV2 exhibit biennial peaks in the fall of odd- and even-numbered years, respectively; whereas HPIV3 demonstrates annual peaks during the summer months [4]. HPIV4 follows a fall–winter seasonal pattern, although data on its circulation remain limited [4]. Given that seasonal activity can vary across different years, ongoing surveillance is crucial for a more comprehensive understanding of these dynamic circulation patterns and their implications for public health [5].

In developed countries, the severity associated with HPIV2 is generally mild, with critical cases primarily occurring among young children, immunocompromised individuals, and the elderly [4,6]. In contrast, in developing regions, the impact is particularly severe among preschool-aged children; with an elevated risk of mortality due to complications such as secondary bacterial infections following HPIV2 infection [7]. Despite the significant disease burden, treatment for HPIV2 primarily focuses on supportive care to alleviate symptoms, as neither specific antiviral agents nor vaccines are currently available. Thus, HPIV2 infection represents a significant public health concern.

HPIV2 has a genome of approximately 15,600 bp, encoding six main structural proteins, including nucleocapsid (N), phosphoprotein (P), matrix (M), hemagglutinin-neuraminidase (HN), fusion (F), and large RNA polymerase (L) [1,8]. Notably, HN and F proteins are crucial for the capacity of the virus to infect host cells. The initial step in HPIV pathogenesis involves viral attachment and fusion with the host cell membrane. The HN protein enables viral attachment to the host epithelial cell by recognizing sialoglycoconjugate receptors [9]. The F protein mediates the fusion of viral and host cell membranes by undergoing a conformational change from a trimeric prefusion form to a trimeric postfusion form, along with energy transfer, allowing the viral nucleocapsid to enter the host cell cytoplasm [10,11].

A previous study showed that HN and F proteins accumulated mutations over time, even within strains from similar geographical areas in HPIV1 [12]. These mutations allow the virus to adapt to different immune pressures, possibly aiding in immune evasion and transmission across hosts. Notably, the F protein is a primary target for neutralizing antibodies in HPIVs, with mutations affecting antibody binding and neutralization potency [13,14,15]. Given these critical functions, both the *HN* and *F* genes are key determinants of viral infectivity, host adaptation, and immune escape. Thus, analyzing the evolutionary dynamics of HPIVs through the *HN* and *F* genes provides valuable insights into their antigenic diversity, genetic variability, and potential implications for vaccine design. As a result, numerous molecular epidemiological and evolutionary studies have focused on these genes to better understand the evolution and transmission of HPIVs [16,17,18,19,20,21]. In a phylogenetic analysis of 15 HPIV2 strains conducted by Terrier et al. in 2008, the HPIV2 *HN* genes were classified into two clusters [17]. Subsequent to advancements in bioinformatics and the progress and widespread adoption of sequencing technologies, HPIV2 has been further classified into two clusters, five lineages, and four sublineages based on the *HN* gene [18]. In contrast, a molecular evolutionary analysis of the HPIV2 *F* gene collected comprehensively has not yet been performed, leaving its evolutionary trajectory poorly understood. Therefore, we analyzed the evolution, phylodynamics, and conformational epitopes of HPIV2 *F* gene sequences collected from different regions of the world.

## 2. Materials and Methods

### 2.1. Strain Selection

We comprehensively collected the full-length coding region of the HPIV2 *F* gene (positions 4789–6444; 1656 nt for HPIV2 strain; NCBI Reference Sequence: NC_003443) from NCBI Virus (https://www.ncbi.nlm.nih.gov/labs/virus/vssi/#/) on 7 August 2023 for molecular evolutionary analysis. Additionally, strains with ambiguous sequences in the *F* gene (e.g., N, Y, R, or V) or unclear collection dates or regions were excluded. Strains with 100% sequence identity were also omitted from the dataset using Clustal Omega (https://www.ebi.ac.uk/jdispatcher/msa/clustalo) on 7 August 2023 [22]. This filtering yielded a final dataset of 102 strains for evolutionary analysis of the HPIV2 *F* gene. These strains were isolated or detected in eight countries between 1955 and 2021. Further details on the strains used in this study are provided in Supplementary Table S1.

### 2.2. Time-Scaled Phylogenetic Tree

To investigate the molecular evolution of the HPIV2 *F* gene, we constructed a time-scaled phylogenetic tree using the Bayesian Markov chain Monte Carlo (BMCMC) method in BEAST (version 2.7.6) [23]. First, we employed the jModelTest2 (version 2.1.10) [24] program to identify appropriate substitution models. Next, the path-sampling/stepping-stone sampling marginal likelihood estimation method was used to determine the best of four clock models (strict clock, exponential relaxed clock, relaxed clock log normal, and random local clock) and the two prior tree models (coalescent constant population and coalescent exponential population). For the BMCMC analysis of all strains, the TPM1uf + G substitution model, random local clock model, and coalescent constant from the tree prior models were selected. A BMCMC tree was then constructed in BEAST2 software based on the obtained strains and selected models. The MCMC chains were run for 300 million steps, with sampling performed every 1000 steps. Effective sample sizes (ESS) were assessed using Tracer (version 1.7.2) [25], with values above 200 deemed acceptable to ensure convergence. After a 15% burn-in, the maximum clade credibility tree was generated using TreeAnnotator version 2.7.6 implemented in BEAST package. Subsequently, the BMCMC tree was visualized by FigTree version 1.4.0 (http://tree.bio.ed.ac.uk/software/figtree/), with branch support provided by 95% highest posterior densities (HPDs). Each cluster identified by the BMCMC tree was subsequently labeled as cluster 1 and cluster 2. Details of parameters used in the BMCMC analyses are shown in Supplementary Table S2.

### 2.3. Genome Population Dynamics and Estimation of Evolutionary Rate

To evaluate the past genome population dynamics of the total and each cluster strain, the effective population sizes were analyzed using Bayesian skyline plot (BSP) generated with BEAST package. The best substitution and clock models were selected, as described above. The MCMC chains of total strain consisted of 100,000,000 steps with sampling every 1000 steps. The visualization of BSPs was presented by Tracer. Moreover, we estimated the molecular evolutionary rate of the total and each cluster strain using Tracer. Statistical analyses were conducted using an unpaired *t*-test in EZR [26]. Statistical significance was defined as *p* < 0.05. The detailed parameters of the BSP analyses are provided in Supplemental Table S2.

### 2.4. Phylogenetic Distance Calculation

To assess the HPIV2 *F* gene diversity, we calculated the phylogenetic distances among the present strains using the Maximum Likelihood (ML) tree in the Patristic program [27]. We conducted the ML phylogenetic analysis utilizing IQ-TREE version 2.2.2.6 with Model Finder, ultrafast bootstrap test parameters, and the SH-like approximate likelihood ratio test [28]. Differences in the data between clusters were compared using an unpaired t-test in EZR. The level of statistical significance was set at *p* < 0.05.

### 2.5. Selective Pressure Analyses

To assume the positive and negative selection sites of the HPIV2 F protein, we computed non-synonymous (*d*N) and synonymous (*d*S) substitution rates using the Datamonkey web server (http://www.datamonkey.org/) on 24 July 2024 [29]. Positive (*d*N/*d*S > 1) and negative (*d*N/*d*S < 1) selection sites were assessed using four methods: Single-Likelihood Ancestor Counting (SLAC), Fixed-Effects Likelihood (FEL), Internal Fixed-Effects Likelihood (IFEL), and Fast Unconstrained Bayesian Approximation (FUBAR). The determination of positive and negative selection was performed based on *p* < 0.05 for SLAC, FEL, and IFEL; and on posterior probabilities > 0.9 for FUBAR.

### 2.6. Structure Modeling

Experimentally determined three-dimensional (3D) structure of the HPIV2 F protein was not available. Hence, we constructed the 3D structure models of HPIV2 F protein of the representative strains from each cluster (prototype: GenBank Accesion Number NC_003443; cluster 1: AF533010; cluster 2: AF533011) using LocalColabFold version 1.5.3 [30]. Terminal regions (3′ and 5′ ends) are frequently exposed and dynamic, which contributes to lower predictive accuracy and greater structural variability in AlphaFold models [31,32]. Thus, we omitted the sequences outside the 3D structural domain of the template HPIV5 F protein (PDBID: 2B9B) from the modeling [33]. The template structure was determined based on sequence identity using the BLAST (https://blast.ncbi.nlm.nih.gov/Blast.cgi) with the PDB on 22 July 2024 [34]. The AMBER force field and structural templates were utilized to generate models. The optimal model was selected based on the predicted Local Distance Difference Test (pLDDT) score, Template Modeling (TM)-score, and Root Mean Square Deviation (RMSD). The best model for each cluster was visualized using PyMOL 3.0.3 (https://www.pymol.org/).

### 2.7. Conformational B-Cell Epitope Prediction

To analyze the human immune pressure on the HPIV2 F protein, the conformational epitopes were predicted using SEPPA 3.0 with a cutoff value of 0.064 on 22 October 2024 [35]. Regions with more than three consecutive amino acids were identified as conformational epitopes and mapped onto the constructed HPIV2 F protein.

## 3. Results

### 3.1. Time-Scaled Phylogenetic and Evolutionary Analyses

**Figure 1:**
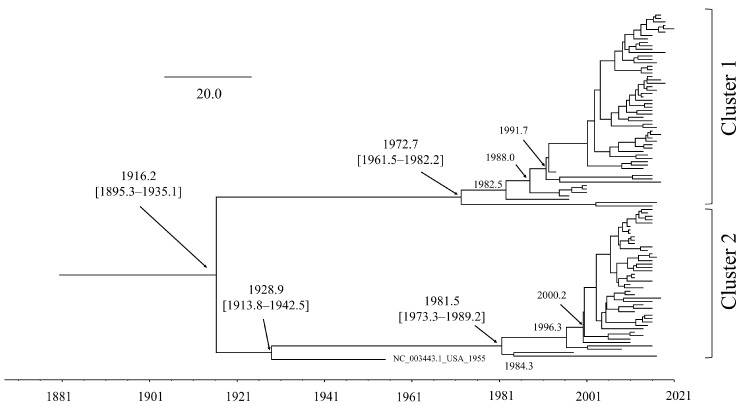
Time-scaled phylogenetic tree of the present strains in *F* gene using the BMCMC method. The scale bar shows time. The number in parentheses represents the 95% HPD interval.

A time-scaled phylogenetic tree of the HPIV2 *F* gene was constructed using the BMCMC method (Figure 1), illustrating the estimated emergence and divergence times of the viral strains. The most recent common ancestor of the present strain was estimated to have diverged around 1916 [mean; 95% highest posterior density (HPD), 1895.3–1935.1], subsequently giving rise to two distinct clusters (clusters 1 and 2). The common ancestor of the strains in cluster 1 is estimated to have emerged around 1972 (mean; 95% HPD interval: 1961.5–1982.2). Cluster 1 strains are widely distributed and form multiple distinct subclusters. Similarly, the common ancestor of the reference strain (NC_003443) and cluster 2 strains likely diverged around 1928 (mean; 95% HPD interval: 1913.8–1942.5). No regional or country-based bias was observed in the distribution of cluster 1 or cluster 2 strains (Supplementary Table S1).

### 3.2. Time-Scaled Genome Population Dynamics

**Figure 2:**
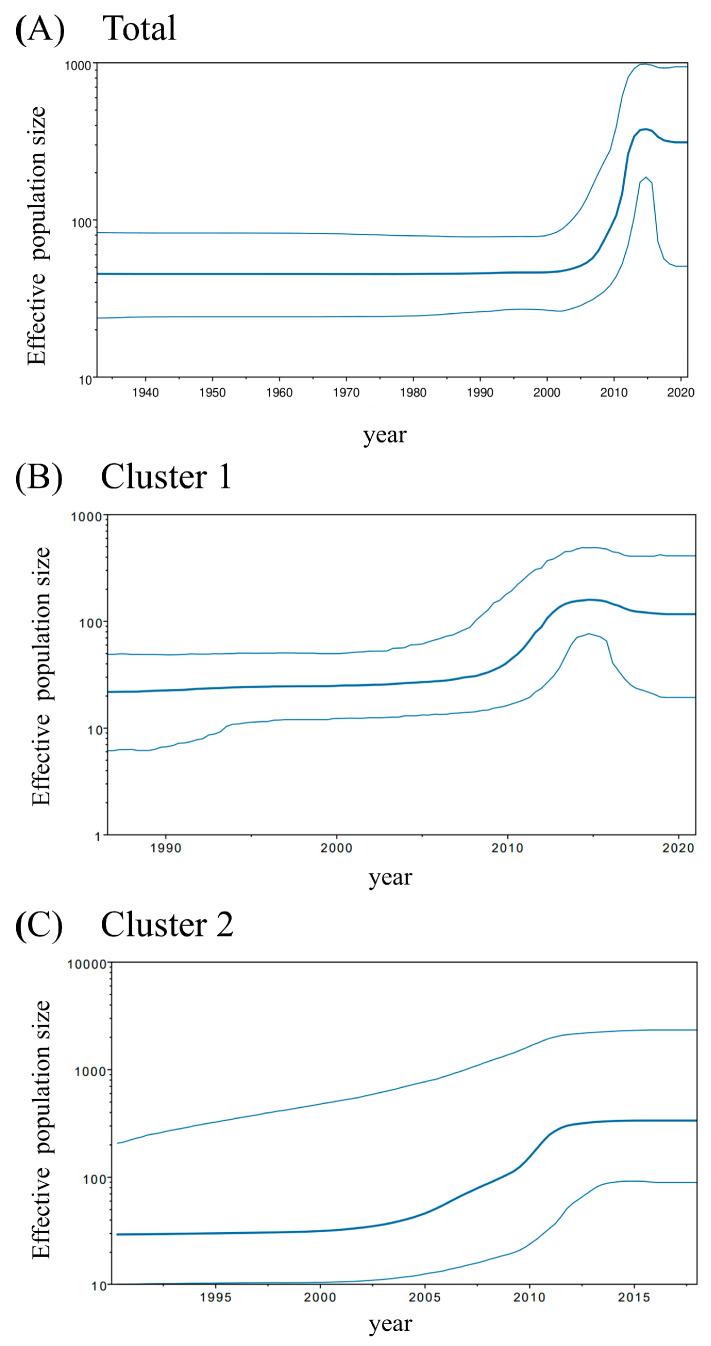
Plots of BSP analyses for the HPIV2 *F* gene. Each panel shows the phylodynamics of all strains (**A**), cluster 1 (**B**), and cluster 2 (**C**). The *y*- and *x*-axes indicate the effective population size and the time in years, respectively. The solid and thin blue lines indicate the mean posterior value and the 95% HPD intervals, respectively.

The phylodynamics of the HPIV2 *F* gene were analyzed using the BSP method (Figure 2), which revealed changes in time-scaled genome population sizes. In all analyzed strains, the genome population size increased significantly from approximately 2005 to 2015, followed by a slight decrease thereafter (Figure 2A). The strain belonging to cluster 1 exhibited a marked increase in genome population size from approximately 2010 to 2015, whereas the strain in cluster 2 showed a gradual increase after 2003 and remained constant from around 2012 (Figure 2B,C). These results suggest that variations in effective population size are cluster-specific; however, the recent increase in population size observed in cluster 1 may reflect the overall phylodynamic trends of all HPIV2 strains.

### 3.3. Estimated Evolutionary Rate

The Bayesian analysis showed that the molecular evolutionary rate of the *F* gene among all HPIV2 strains was calculated as approximately 4.20 × 10^−4^ substitutions/site/year (mean; 95% HPD, 3.31 × 10^−4^ to 4.51 × 10^−4^). The evolutionary rate of strains belonging to cluster 1 (mean, 4.23 × 10^−4^; 95% HPD, 2.97 × 10^−4^ to 5.50 × 10^−4^ substitutions/site/year) was significantly higher than that of strains belonging to cluster 2 (mean, 2.47 × 10^−4^; 95% HPD, 8.57 × 10^−5^ to 4.16 × 10^−4^ substitutions/site/year) (*p* < 0.001). These results indicate that the strains belonging to clusters 1 and 2 have evolved independently at distinct evolutionary rates.

### 3.4. Phylogenetic Distance

**Figure 3:**
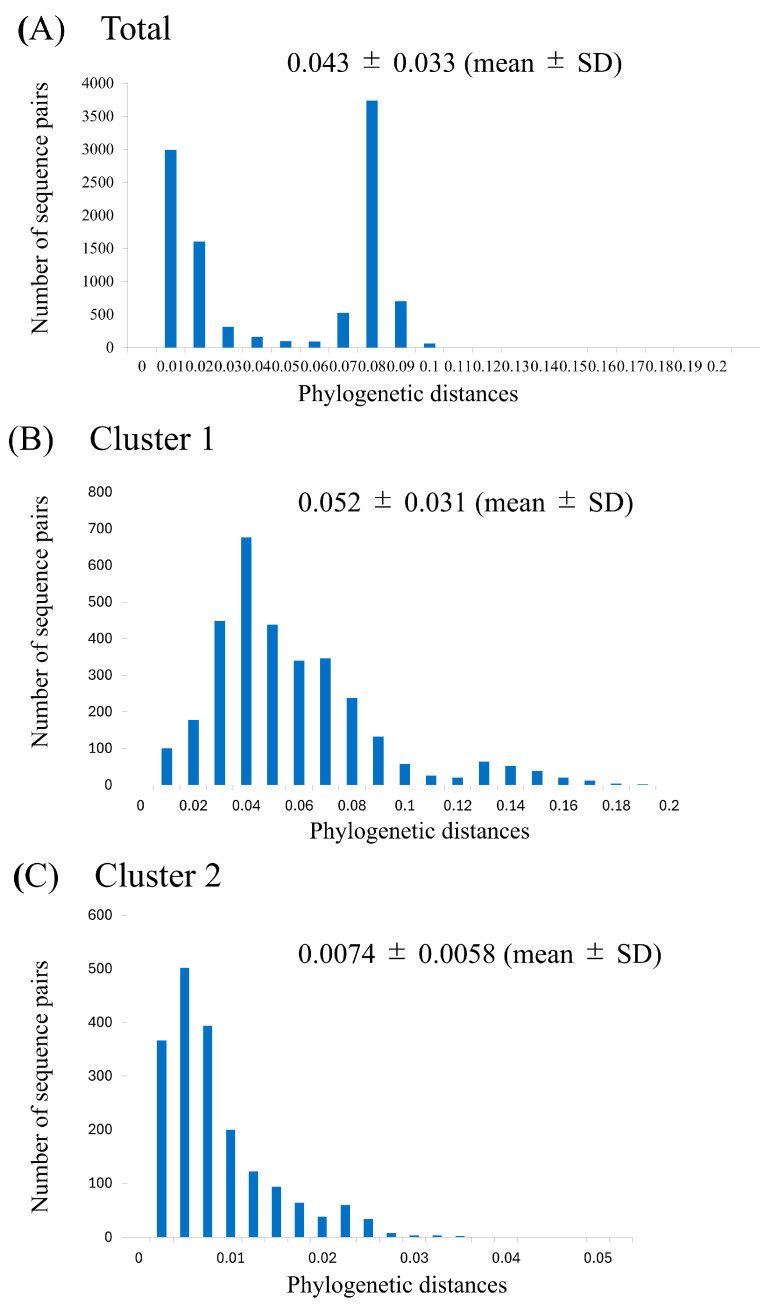
Phylogenetic distances for the HPIV2 *F* gene of all strains (**A**), cluster 1 (**B**), and cluster 2 (**C**). The *y*- and *x*-axes show the number of sequence pairs and phylogenetic distances, respectively.

Phylogenetic distances of the *F* gene were calculated using an ML-based phylogenetic tree (Figure 3), providing an assessment of genetic divergence. A histogram of the distances among all strains showed a bimodal distribution, with the phylogenetic distance calculated as 0.043 ± 0.033 (mean ± SD) (Figure 3A). The phylogenetic distances of the strains in clusters 1 and 2 were 0.052 ± 0.031 and 0.0074 ± 0.0058, respectively (Figure 3B,C); indicating that the strains in cluster 1 exhibited significantly greater genetic divergence than those in cluster 2 (*p* < 0.001).

### 3.5. Positive and Negative Selection Sites

**Figure 4:**
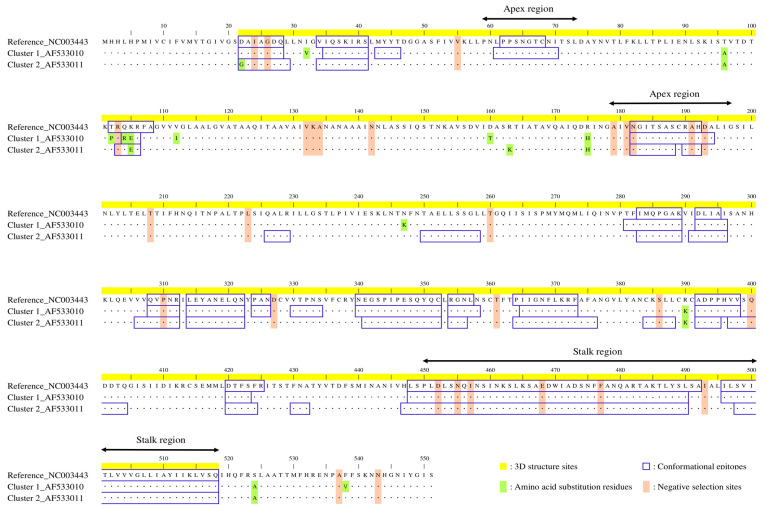
Amino acid sequence alignment of the HPIV2 F proteins. The amino acid residue numbers used for constructing HPIV2 F protein 3D structure are highlighted in yellow. Blue line boxes in the sequence indicate conformational epitopes. The amino acid substitution and negative selection sites are highlighted in light green and light brown, respectively.

The *d*N/*d*S substitution ratio was estimated using the DataMonkey web server to assess selection pressure. This analysis identified positive selection, which favors advantageous mutations; and negative selection, which eliminates deleterious mutations. FUBAR identified a single residue, amino acid 104 (aa104), as a site under positive selection. However, comparison of the four methods—SLAC, FEL, IFEL, and FUBAR—revealed no consistently identified sites under positive selection, suggesting limited evidence for selection at any specific amino acid site. In contrast, thirty residues were consistently identified as being under negative selection by all four methods (Figure 4). These sites under negative selection were distributed irregularly across the HPIV2 F protein. Similarly, several amino acid substitutions were scattered throughout the F protein.

### 3.6. 3D Structure and Conformational Epitopes

**Figure 5:**
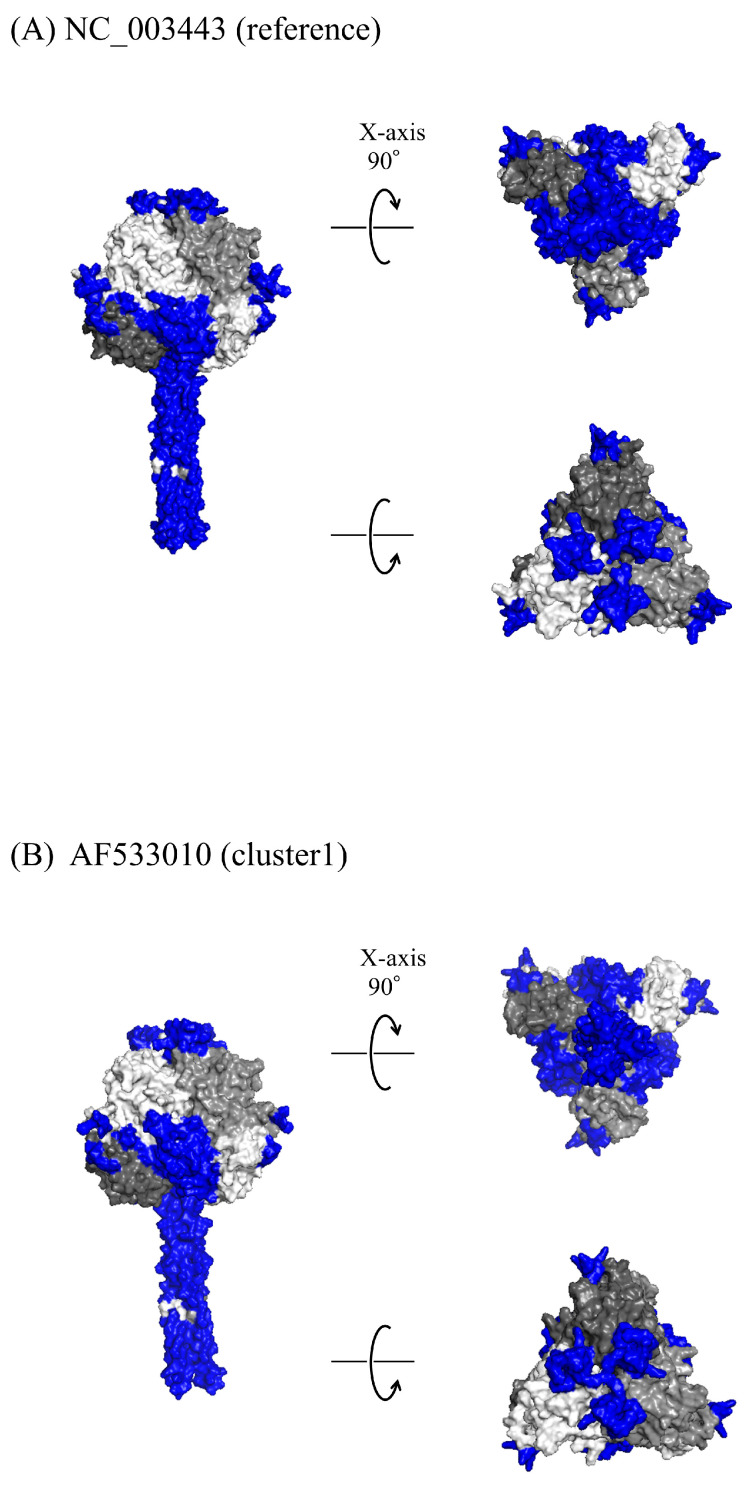

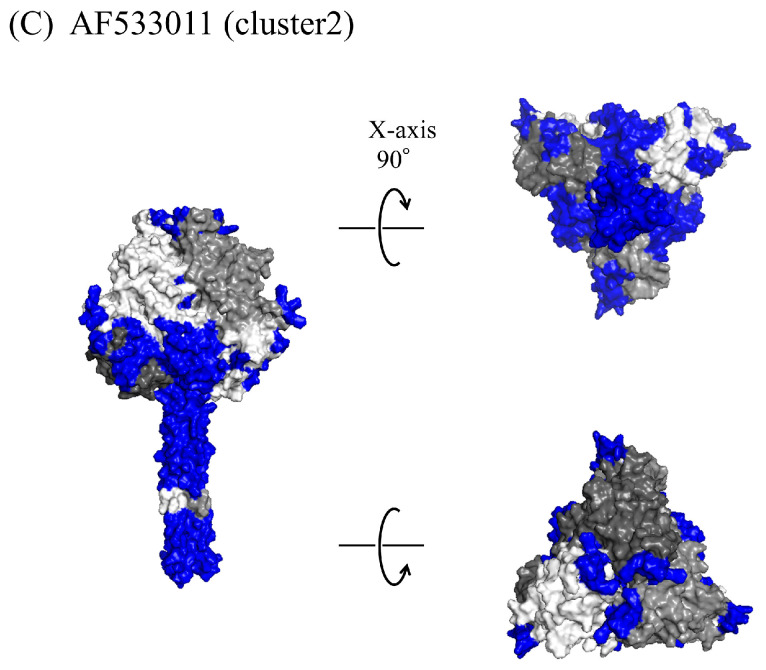
Structural models of the HPIV2 F protein. The figures depict the F protein structures of the prototype strain (**A**), cluster 1 strain (AF533010) (**B**), and cluster 2 strain (AF533011) (**C**). The chains of the trimeric structures are represented in light gray, dim gray, and black. Conformational epitopes are shown in blue.

To explore the immunogenic properties of the HPIV2 F protein, its 3D structure was constructed, and conformational epitopes were mapped (Figure 5A–C). The constructed F trimeric protein presented a “tree-like” appearance with a globular head and a stalk region, consistent across the prototype and representative strains of each cluster. However, the precise amino acid boundaries of the globular head and stalk regions in the HPIV2 F protein have not been clearly established. Thus, based on the structural template of the HPIV5 F protein (PDB ID: 2B9B), we defined the stalk region as spanning amino acids 450–518, while the remaining portion was designated as the globular head region (Figure 4). In addition, within the globular head region, amino acids 58–73 and 179–197 were defined as the apex region (Figure 4). The conformational epitope sites were predominantly located in the apex and stalk regions; a pattern that was consistent across the strains. However, regions unique to specific clusters were also identified, including residues aa330–334 in cluster 1 and residues aa226–229, aa250–258, and aa384–388 in cluster 2 (Figure 4 and Figure 5). All of these epitope sites are situated within the globular head domain, excluding the apex region. Furthermore, residues aa61–70 within the apex region were predicted as an epitope in cluster 1, but not in cluster 2 (Figure 4 and Figure 5). Although amino acid substitutions in the epitope region were relatively rare, specific mutations were observed, including D22G in cluster 2; T102P and Q104R in cluster 1; and K105E in both clusters (Figure 4). All of these mutations were located within the globular head domain, rather than the apex. With the exception of T102P, these mutations were predicted to be part of epitope sites in both clusters. Details of the predicted conformational epitope sites are shown in Supplementary Table S3.

## 4. Discussion

HPIV2 is a major cause of respiratory infections, particularly in young children, immunocompromised individuals, and the elderly. Despite its clinical importance, no licensed vaccines or antiviral treatments for HPIV2 are currently available, leaving supportive care as the main option for managing infections [36]. Understanding the evolutionary dynamics of the *F* gene is essential for developing effective countermeasures, as it provides insights into the virus’s adaptability and antigenic diversity. This study leveraged bioinformatics techniques to investigate the evolutionary trajectory of the *F* gene, shedding light on the temporal dynamics, selective pressures, and structural-functional constraints that govern its evolution. Our phylogenetic analysis revealed that the HPIV2 *F* gene diverged into two major clusters, with their most recent common ancestor estimated to have existed around the early 20th century, consistent with a previous evolutionary study of the HPIV2 *HN* gene [18]. Furthermore, when comparing strains common to both studies, we found that all strains classified as cluster 1 in our study correspond to cluster II in the HN evolutionary study; while those categorized as cluster 2 in our study align with cluster I in the HN study. Cluster 1, with its higher evolutionary rate, underwent more significant genetic diversification compared to cluster 2. The time-scaled phylogenetic tree further highlights the sustained evolution of both clusters, with independent lineages exhibiting distinct adaptive strategies.

The BSP analyses revealed changes in the effective genome population size of HPIV2. The genome population size for the HPIV2 *F* gene exhibited significant expansion between 2005 and 2015, coinciding with increased global reporting of respiratory infections [37,38]. This expansion may correlate with enhanced diagnostic capabilities and surveillance efforts, rather than intrinsic viral factors alone. For cluster 2, a steady but less pronounced population increase was observed, followed by stabilization, suggesting a more geographically restricted or epidemiologically stable lineage. These variations in effective population sizes may reflect differences in transmission dynamics, immunological pressures, and ecological factors [39,40,41]. The larger fluctuations in cluster 1 also underscore its potential for broader dissemination and adaptability in diverse host populations. Indeed, cluster 1 exhibited a higher evolutionary rate and greater genetic diversity compared to cluster 2. With respect to potential effects on virulence and transmission, the increased genetic diversity observed in cluster 1 may contribute to the emergence of variants with enhanced viral fitness, potentially influencing disease severity and infectivity. Mutations occurring in antigenic sites or functional domains of the F protein could modify host cell entry efficiency, immune evasion mechanisms, or replication capacity, thereby impacting clinical outcomes and facilitating more efficient spread across diverse populations and geographic regions. However, no specific country-based bias was observed in the distribution of strains belonging to these clusters. This lack of geographic bias suggests that global dispersal and transmission dynamics, rather than localized environmental factors, play a more significant role in shaping the evolutionary patterns of HPIV2. Nevertheless, as this study evaluated regional differences only at the national level, it remains possible that more specific factors, such as strains originating from particular regions or medical facilities, may have influenced the results.

The evolutionary rate of the HPIV2 *F* gene, estimated at 4.20 × 10^−4^ substitutions/site/year, reflects the interplay between functional constraints and adaptive pressures. Comparisons with other paramyxoviruses highlight shared and distinct evolutionary patterns. The HPIV4 and mumps virus (MuV) *F* genes evolve at a similar rate of 4.41 × 10^−4^ and 5.0 × 10^−4^ substitutions/site/year, respectively [21,42]. In contrast, human respiroviruses, such as HPIV1 and HPIV3, exhibit higher *F* gene evolutionary rates of 8.50 × 10^−4^ and 9.40 × 10^−4^ substitutions/site/year, respectively [16,20]. These rates may reflect stronger immune selection pressures and broader ecological adaptations. Zoonotic and avian paramyxoviruses, such as avian paramyxovirus 4 (APMV-4), have higher *F* gene evolutionary rates, with APMV-4 evolving at 1.29 × 10^−3^ substitutions/site/year [43]. This accelerated rate is likely driven by cross-species transmission and the need for rapid adaptation to diverse hosts [44,45]. These comparisons demonstrate that the molecular evolutionary rate of the HPIV2 *F* gene is placed in an intermediate position among paramyxoviruses. Its slower evolution compared to zoonotic and avian viruses reflects the stability of its human-specific ecological niche. However, it is worth noting that even subtle evolutionary changes in the *F* gene can have profound implications for antigenic properties and immune recognition [46].

The F protein plays a central role in HPIV2 infectivity by facilitating the fusion of viral and host cell membranes. As such, it is subject to significant evolutionary constraints that maintain its structural and functional integrity. Our selective pressure analysis revealed that the *F* gene was predominantly under purifying selection, with negative selection sites distributed throughout the protein. This finding aligns with the critical role of the F protein in viral entry and its conservation across paramyxoviruses [11,47]. In contrast, in cluster 1, an amino acid substitution (V112I) was observed in a functionally important domain such as the fusion peptide region (aa107–131) [48,49]. However, the present analysis did not provide sufficient information to determine the impact of this mutation on membrane fusion. In addition, no robust positive selection sites were identified in this study, suggesting that adaptive changes in the *F* gene are rare. This may be due to the protein’s structural constraints, which limit the range of permissible mutations without compromising function. These findings are consistent with previous studies on other HPIVs and suggest that the conservation of F proteins is a critical factor in determining viral fitness [16,20,21].

Structural modeling of the F protein revealed additional insights into its antigenic landscape, with conformational epitopes predominantly located in the apex and stalk regions. Notably, the apex region has been identified as a key target for neutralizing antibodies in other paramyxoviruses, such as HPIV3 and respiratory syncytial virus (RSV), underscoring its importance for understanding immune interactions and guiding vaccine design [50,51,52,53]. While the immunogenic properties of these regions in HPIV2 remain to be experimentally validated, insights from related paramyxoviruses suggest their role in eliciting robust immune responses. In the present study, the apex region was identified as an epitope shared across clusters, suggesting its potential utility as a target for vaccine development or therapeutic antibody design. However, in cluster 2, certain residues within the apex region were not predicted as epitopes, which could affect antibody binding efficacy.

This study demonstrated that—while amino acid substitutions within the epitope region were relatively uncommon—specific mutations did occur, particularly within the globular head domain of the HPIV2 F protein. The identified amino acid changes did not substantially alter the epitope regions. However, considering the distinction between antigenicity—the ability of an epitope to be recognized by antibodies—and immunogenicity—the capacity to elicit a functional immune response leading to viral clearance—the precise effect of these mutations on the binding affinity of neutralizing antibodies remains unclear [54]. Notably, several of the observed mutations cluster near the fusion peptide region of the HPIV2 F protein (aa107–131) [48,49], suggesting that this region may serve as a potential epitope targeted by neutralizing antibodies. Thus, amino acid substitutions within the epitope region—particularly the K105E substitution identified in both clusters—may play a crucial role in modulating immunogenicity.

The findings of this study provide valuable insights that contribute to the development of effective vaccines. The present study indicated that HPIV2 has the potential to undergo limited antigenic drift, despite the strong purifying selection acting on its *F* gene. These antigenic changes must be considered in vaccine design, as a vaccine targeting a single strain may not fully neutralize emerging variants with altered surface epitopes. Notably, the study identified slight differences in evolutionary rates between the two major phylogenetic clusters, with cluster 1 exhibiting a higher substitution rate. This suggests that viruses within cluster 1 may accumulate antigenic mutations at a relatively faster rate. A higher evolutionary rate could reduce the duration of vaccine strain effectiveness, as immunity induced by a fixed antigen may be circumvented more rapidly by an evolving viral lineage. Therefore, the development of an effective HPIV2 vaccine should prioritize broad coverage strategies. This may include targeting conserved regions of the F protein or employing a multivalent approach incorporating representative strains from both clusters to enhance cross-protective immunity. Additionally, strengthening epidemiological surveillance is crucial for the early detection of significant viral variants and for generating data that can inform vaccine updates [55,56]. By integrating molecular surveillance with immunological data, public health authorities can better anticipate HPIV2’s evolutionary trends and implement targeted interventions.

This study has several limitations. First, the dataset included publicly available sequences, potentially underrepresenting certain regions or time periods. Second, the lack of clinical and epidemiological data precludes direct correlations between genetic variation and disease severity. Finally, the present study lacks experimental analyses. While epitope prediction and structural modeling provide valuable insights, functional studies such as ELISA, neutralization tests, and site-directed mutagenesis are necessary to confirm the impact of these mutations on immune recognition and viral fitness. Furthermore, molecular dynamics simulations could offer additional insights into protein stability and conformational dynamics. Therefore, future studies integrating both computational and experimental approaches will be necessary to achieve a more comprehensive understanding of HPIV2 F protein evolution and immune evasion mechanisms.

## 5. Conclusions

This study provides a comprehensive analysis of the molecular evolution of the HPIV2 *F* gene, highlighting its divergence into distinct clusters, the selective pressures shaping its evolution, and its structural-functional constraints. By integrating phylogenetic, phylodynamic, and structural analyses, this study deepens our understanding of HPIV2’s evolutionary landscape and provides a foundation for future research to mitigate its public health impact.

## Data Availability

The original contributions presented in this study are included in the article/Supplementary Material. Further inquiries can be directed to the corresponding authors.

## Supplementary Materials

The following supporting information can be downloaded at: https://www.mdpi.com/article/10.3390/microorganisms13020399/s1, Table S1: Detailed data on the strains used in this study; Table S2: Parameters used in the BMCMC analyses; Table S3: Conformational epitopes of the HPIV2 F protein.
